# Inflammation Affects Liver Function and the Metabolism of Voriconazole to Voriconazole-N-Oxide in Adult and Elderly Patients

**DOI:** 10.3389/fphar.2022.835871

**Published:** 2022-04-06

**Authors:** Zaiming Liang, Mingjie Yu, Zhirui Liu, Fang Liu, Changsheng Jia, Lirong Xiong, Qing Dai, Shiwei Qin, Lin Cheng, Fengjun Sun

**Affiliations:** Department of Pharmacy, The First Affiliated Hospital of Third Military Medical University (Army Medical University), Chongqing, China

**Keywords:** voriconazole, inflammation, voriconazole-N-oxide, invasive fungal infections, plasma concentration, therapeutic drug monitoring

## Abstract

**Background:** The inner association of inflammation with voriconazole (VCZ) metabolism has not been fully investigated. We intend to investigate the effects of inflammation on liver function, VCZ trough concentration (C_0_), C_0_/dose ratio and the ratio of VCZ to VCZ-N-oxide concentration (C_0_/C_N_) in adult and elderly patients.

**Methods:** A single-center retrospective study was conducted among patients who were treated in our hospital between January 2018 and December 2021. For each eligible patient, demographic details, medical history, laboratory parameters, procalcitonin (PCT), C reactive protein (CRP), and interleukin-6 (IL-6) were collected from the medical chart. VCZ C_N_, TNF-α, IL-1β, IL-8, and IL-10 concentrations were detected in blood samples.

**Results:** A total of 356 patients were included in our study, with 195 patients in the adult cohort (<60 years) and 161 patients in the elderly cohort (≥60 years). In adult patients, CRP and IL-8 levels showed moderate association with VCZ C_0_/C_N_ ratio (CRP: r = 0.512, *p* < 0.001; IL-8: r = 0.476, *p* = 0.002). IL-6 level shallowly associated with VCZ C_0_/C_N_ ratio both in adult and elderly patients (r = 0.355, *p* = 0.003; r = 0.386, *p* = 0.001). A significantly higher VCZ C_0_, C_0_/dose ratio and C_0_/C_N_ ratio was observed in adult patients with severe inflammation compared with patients with moderate inflammation and no to mild inflammation, as reflected by PCT levels (*p* < 0.05). However, there was no significant difference observed among different inflammation degrees in elderly patients. Lower albumin (AL) and higher total bilirubin (TBIL) were observed along with the degree of inflammation in both adult and elderly patients, as reflected by CRP and PCT levels (*p* < 0.05).

**Conclusion:** Inflammation may affect the metabolism of VCZ to VCZ-N-oxide both in adult and elderly patients, and decreased plasma AL levels and increased TBIL levels under inflammatory conditions may also alter VCZ metabolism.

## Introduction

Invasive fungal infections are common complications in immunosuppressed patients with high morbidity and mortality, such as patients with hematologic malignancy, solid organ transplant patients, and intensive care unit patients (Jenks et al., 2020). The incidence of invasive fungal infections in elderly patients is also obvious due to their decreased physiological defense function and immune function and complicated chronic diseases such as hypertension, diabetes, coronary atherosclerotic heart disease, etc. Voriconazole (VCZ) is a triazole antifungal agent widely used in patients with invasive *Aspergillus* or *Candida* infection (Patterson et al., 2016). It can be used as prophylactic or therapeutic. However, the therapy range of VCZ is narrow; in Europe, it is 1.0–5.5 μg/ml, and in China, it is 0.5–5.0 μg/ml (Chen et al., 2018; Ullmann et al., 2018). The metabolism of VCZ exhibits nonlinear pharmacokinetic characteristics with highly individual variability (Theuretzbacher et al., 2006). Many factors have been reported to contribute to the concentration variability of VCZ, including age, sex, drug-drug interactions, liver dysfunction, serum total bilirubin (TBIL), albumin (AL), cytochrome P450 (CYP) 2C19 gene polymorphism, and inflammation (Theuretzbacher et al., 2006; Bruggemann et al., 2009; Weiss et al., 2009; Veringa et al., 2017; Vreugdenhil et al., 2018; Xu et al., 2018; Shang et al., 2020).

Recently, much work has been done to explore the association of inflammation, as reflected by C reactive protein (CRP), interleukin-6 (IL-6), and IL-8, with VCZ concentration in transplantation patients, patients with blood diseases, and critically ill patients (van Wanrooy et al., 2014; Vreugdenhil et al., 2018; Gautier-Veyret et al., 2019). In our previous study, we identified that procalcitonin (PCT) is an independent factor affecting the VCZ trough concentration (C_0_) in elderly patients (Cheng et al., 2019). In the case of infection, immune cells release tumor necrosis factor (TNF-α), IL-1, IL-6, IL-8 and other signaling molecules, causing a systemic inflammatory response and stimulating different tissues (kidney, adipose tissue, lung, and liver) to secrete PCT, and their blood concentrations increase (Maruna et al., 2000). Although CRP, IL-6, and IL-8 were associated with VCZ C_0_ (Vreugdenhil et al., 2018) and CRP was correlated with the metabolism of VCZ in hematological patients (major adults) (Veringa et al., 2017), the effect of IL-6 and IL-8 on VCZ metabolism in adult and elderly patients is still unknown.

The VCZ C_0_ in the elderly cohort were significantly higher than those in the adult cohort at the same normal dosage, and the proportion of VCZ C_0_ > 5.0 μg/ml was 2.3 fold in the elderly cohort than that in the adult cohort (35.3% vs. 15.4%) (Cheng et al., 2019). In the previous study, we also found that for patients with CYP2C19 normal metaboliser, the VCZ C_0_, C_0_/dose ratio and the ratio of VCZ to VCZ-N-oxide concentration (C_0_/C_N_) were significantly higher in the elderly cohort than those in the adult cohort (Shang et al., 2020). The adverse effects of VCZ were the main cause of drug discontinuation, and thus lead to high mortality, which was associated with its trough concentration (Chan et al., 2020; Tang et al., 2020). Infection, chronic inflammation induced by chronic disease condition, and inflammation induced by the treatment of immunosuppressants were commonly existed both in adult and elderly patients with invasive fungal infections. In addition, liver function has been generally accepted to affect the concentration of VCZ, and the liver function of elderly patients is impaired, with decreased expression of albumin (AL) and CYP enzymes and increased bilirubin concentrations (Schmucker, 2005; Dong et al., 2010), which may also affect VCZ metabolism.

VCZ metabolism is mainly mediated by the drug-metabolizing enzymes CYP2C19 and CYP3A4 in the liver, and the main metabolite in plasma is VCZ-N-oxide (accounting for 72%), with almost no activity (Hyland et al., 2003). To confirm the effect of inflammation on VCZ metabolism, the ratio of VCZ C_0_/C_N_ might provide more information. The association of VCZ C_0_/C_N_ with inflammation was not fully investigated in elderly patients. Combined with the impaired liver function and chronic inflammation of elderly patients, we intend to investigate the influence of inflammation on liver function and VCZ metabolism in elderly patients, and take the results of adults as control, so as to provide evidence of the rational use of VCZ in the elderly.

## Materials and Methods

### Patients and Study Design

A single center retrospective study was conducted. The patients were treated at Southwest Hospital, Chongqing, China between January 2018 and December 2021. Patients who met the following inclusion criteria were included: 1) aged ≥18 years; 2) hospitalized patient who had at least one steady-state VCZ C_0_ and CRP, PCT, or IL-6 concentration and liver function measured on the same day; and 3) not concomitantly using a strong inhibitor or inducer of CYP isoenzymes.

### Data Collection

For each eligible patient, the following data were collected from the medical chart: 1) demographic details, including age, sex, and body weight; 2) medical history, including underlying disease, type of invasive fungal infections, and VCZ treatment regimen; 3) laboratory parameters, such as the liver function indices, ALP, ALT, AST, γ-GT, TBIL, and AL; and 4) inflammation markers, including PCT, CRP, and IL-6, which were measured routinely.

### VCZ C_0_ and VCZ C_N_ Determination

The VCZ C_0_ was defined as the concentration obtained after 3 days of VCZ therapy with a loading dose of 6 mg/kg by intravenous administration, and maintenance dose of 4 mg/kg by intravenous administration, or sequentially taking VCZ orally (200 mg). The VCZ C_0_ and VCZ C_N_ were measured by liquid chromatography-tandem mass spectrometry (LC–MS/MS) as previously described (Shang et al., 2020).

### Cytokine Concentration Determination

Only discarded blood samples for VCZ C_0_ determination between May 2021 and November 2021 were used. A total of 161 of the VCZ C_0_ values matched with samples that were available for cytokine determination. IL-1β, IL-10, and TNF-α enzyme-linked immune sorbent assay (ELISA) kits were purchased from Jianglai Biological (Shanghai, China). IL-8 ELISA kits were purchased from Elabscience Biotechnology Co., Ltd. Plasma concentrations of the cytokines were measured according to the manufacturer’s instructions.

### Statistical Analysis

IBM SPSS 19.0 (IBM Corp., Armonk, NY, United States) was used to perform the analysis. Categorical data were compared with the chi-squared test or Fisher’s exact test. To explore the possible association of inflammation with VCZ C_0_ and liver function, VCZ C_0_, C_0_/dose ratio, C_0_/C_N_ ratio, and liver function of patients with no to mild inflammation (CRP, ≤40 mg/L; PCT, ≤0.25 ng/ml), with moderate inflammation (CRP, 41–200 mg/L; PCT, 0.25–0.5 ng/ml), and with severe inflammation (CRP, >200 mg/L; PCT, >0.5 ng/ml) were compared using a Kruskal–Wallisone test (three groups) or a Mann–Whitney U test (two groups). Pearson’s correlation test was performed to assess the association of inflammation, reflected by the above inflammatory markers, with VCZ C_0_, C_0_/dose ratio, and C_0_/C_N_ ratio.

## Results

### Patient Characteristics

A total of 356 patients were included in our study, with 195 patients in the adult cohort (<60 years) and 161 patients in the elderly cohort (≥60 years). The baseline patient characteristics are depicted in Table 1. The main baseline diseases in the elderly cohort were hypertension, diabetes mellitus, and coronary atherosclerotic heart disease, while the main baseline diseases in the adult cohort were leukemia and kidney disease. The main infected fungi were *Monilia*, *Saccharomycetes*, and *Aspergillus*.

**TABLE 1:** Baseline characteristics of patients in the two cohorts.

Variable	Adult cohort (*n* = 195)	Elderly cohort (*n* = 161)	*p* value
Sex			0.110
Male [no. (%)]	116 (59.5)	109 (67.7)	
Female [no. (%)]	79 (40.5)	52 (32.3)	
Age (yr)	42.4 ± 12.0	71.9 ± 8.4	0.000
Weight (kg)	60.35 ± 12.05	57.84 ± 11.91	1.651
Underlying diseases			
Leukemia [no. (%)]	77 (39.5)	12 (7.4)	0.000
Hypertension [no. (%)]	41 (21.0)	65 (40.4)	0.000
Diabetes mellitus [no. (%)]	24 (12.3)	49 (30.4)	0.000
Kidney disease [no. (%)]	60 (30.8)	48 (29.8)	0.757
Renal transplant [no. (%)]	8 (4.1)	0	0.025
Liver cancer [no. (%)]	0	4 (2.5)	0.088
Pneumonia [no. (%)]	98 (50.3)	95 (59.0)	0.099
Coronary atherosclerotic heart disease [no. (%)]	1 (0.5)	96 (59.6)	0.000
Fungus category			
Aspergillus [no. (%)]	22 (11.3)	20 (12.4)	0.740
Saccharomycetes [no. (%)]	16 (8.2)	33 (20.5)	0.001
Monilia [no. (%)]	31 (15.9)	31 (19.3)	0.406
Others [no. (%)]	40 (20.5)	34 (21.1)	0.997
Negative [no. (%)]	89 (45.6)	43 (26.7)	0.000
Route of administration			0.362
Intravenous [no. (%)]	142 (72.8)	114 (70.8)	
Oral [no. (%)]	15 (7.7)	8 (5.0)	
Mixed [no. (%)]	38 (19.5)	39 (24.2)	
Daily voriconazole dose (mg/kg)	7.34 ± 1.36	7.33 ± 1.52	0.944

### VCZ C_0_, C_0_/Dose Ratio and C_0_/C_N_ Ratio

The VCZ C_0_, C_0_/dose ratio, and C_0_/C_N_ ratio in the elderly cohort were significantly higher than those in the adult cohort (*p* < 0.05, Table 2).

**TABLE 2:** Laboratory parameters of patients in the two cohorts.

Parameter	*n*	Adult cohort	*n*	Elderly cohort	*p* value
VCZ C_0_ (μg/ml)	298	2.90 (1.50, 4.90)	255	3.60 (2.30, 5.65)	0.002
VCZ C_0_/dose ratio (g/ml)	217	0.42 (0.22, 0.72)	143	0.53 (0.30, 0.81)	0.036
VCZ C_0_/C_N_ ratio	154	1.42 (0.67, 3.63)	130	2.05 (1.11, 3.20)	0.029
Inflammation marker					
CRP (mg/L)	89	42.90 (8.96, 119.55)	41	64.07 (14.50, 131.00)	0.106
PCT (ng/ml)	172	0.29 (0.10, 0.84)	127	0.30 (0.11, 0.91)	0.395
TNF-α (pg/ml)	53	4.59 (3.29, 7.58)	38	27.17 (4.68, 60.36)	0.000
IL-6 (ng/L)	139	26.63 (7.47, 97.30)	119	33.87 (16.08, 75.54)	0.336
IL-8 (pg/ml)	73	17.69 (16.11, 42.80)	88	16.97 (13.79, 37.08)	0.279
IL-10 (pg/ml)	46	30.41 (19.05, 58.30)	41	62.99 (20.48, 116.82)	0.094
IL-1β (pg/ml)	38	4.85 (2.53, 8.25)	33	6.88 (3.76, 16.33)	0.122
Liver function					
ALT (U/L)	193	96.0 (74.0, 151.0)	167	114.0 (79.5, 176.0)	0.056
AST (U/L)	193	18.8 (9.6, 37.5)	167	22.4 (10.85, 41.4)	0.221
γ-GT (U/L)	193	24.2 (16.1, 50.6)	167	34.2 (22.9, 56.9)	0.000
ALP (U/L)	193	73.1 (34.9, 143.9)	167	86.0 (43.8, 174.35)	0.105
TBIL (μmol/L)	193	13.9 (9.2, 24.5)	167	13.3 (9.65, 25.9)	0.777
AL (g/L)	193	32.1 (29.1, 35.5)	167	32 (29.05, 36.55)	0.711

Data are presented as median (interquartile range). VCZ, voriconazole; CRP, C-reactive protein; PCT, procalcitonin; TNF, tumor necrosis factor; IL, interleukin; ALT, alanine aminotransferase; AST, aspartate aminotransferase; γ-GT, γ-glutamyl transferase; ALP, alkaline phosphatase; TBIL, total bilirubin; AL, albumin.

### Determination of Levels of Cytokines, CRP, and PCT

An overview of all measured laboratory parameters can be found in Table 2. The plasma levels of TNF-α and γ-GT in the elderly cohort were significantly higher than those in the adult cohort (*p* < 0.001).

### Correlations Between Inflammatory Markers and VCZ C_0_

**FIGURE 1:**
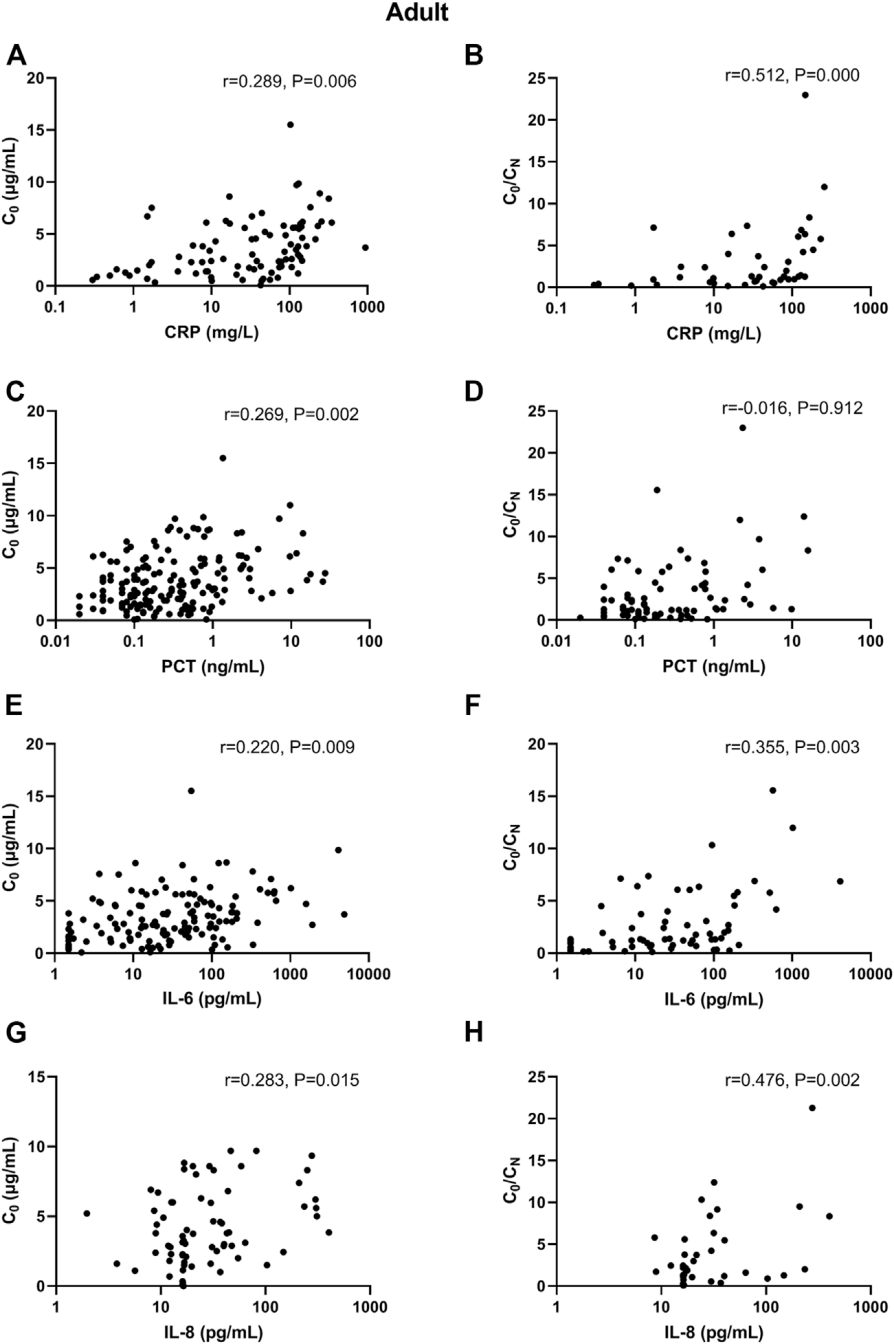
Association of CRP, PCT, IL-6, and IL-8 plasma levels with VCZ C_0_ and VCZ C_0_/C_N_ in adult patients. **(A,B)** The CRP levels were poorly associated with VCZ C_0_ but moderately associated with VCZ C_0_/C_N_ ratio. **(C,D)** The PCT levels were poorly associated with VCZ C_0_ but not associated with VCZ C_0_/C_N_ ratio. **(E,F)** The IL-6 levels were poorly associated with VCZ C_0_ but moderately associated with VCZ C_0_/C_N_ ratio. **(G,H)** The IL-8 levels were poorly associated with VCZ C_0_ but moderately associated with VCZ C_0_/C_N_ ratio.

**FIGURE 2:**
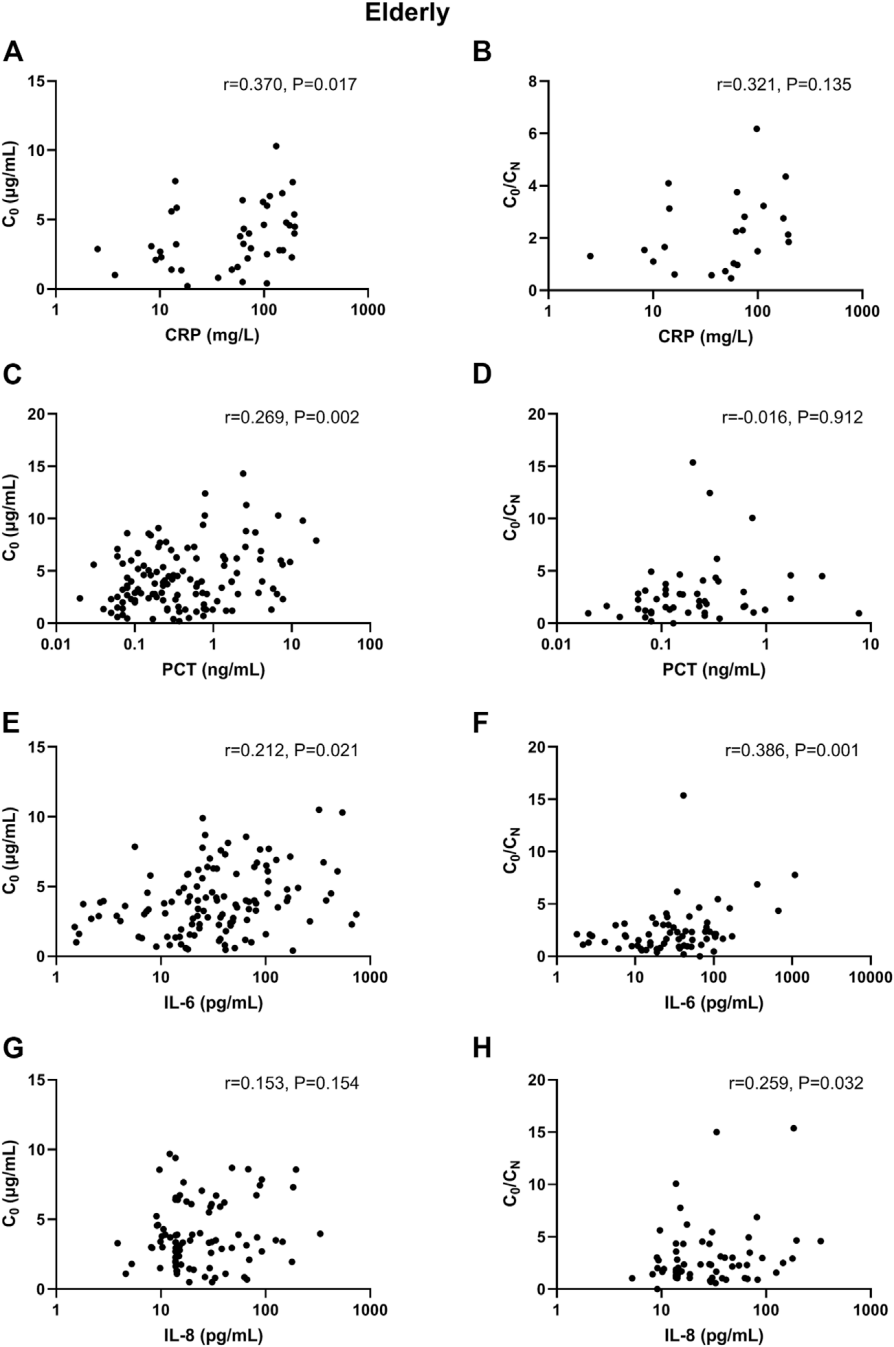
Association of CRP, PCT, IL-6, and IL-8 plasma levels with VCZ C_0_ and VCZ C_0_/C_N_ ratio in elderly patients. **(A,B)** The CRP levels were moderately associated with VCZ C_0_ but not associated with VCZ C_0_/C_N_ ratio. **(C,D)** The PCT levels were poorly associated with VCZ C_0_ but not associated with VCZ C_0/_C_N_ ratio. **(E,F)** The IL-6 levels were poorly associated with VCZ C_0_ but moderately associated with VCZ C_0_/C_N_ ratio. **(G,H)** The IL-8 levels were not positively associated with VCZ C_0_ but were poorly associated VCZ C_0_/C_N_ ratio.

In adult patients, CRP and PCT levels showed poor association with VCZ C_0_, as well as IL-6 and IL-8 level (Figure 1). In elderly patients, only CRP level showed shallow association with VCZ C_0_ (CRP: r = 0.370, *p* = 0.017, 41 observations) (Figure 2).

**FIGURE 3:**
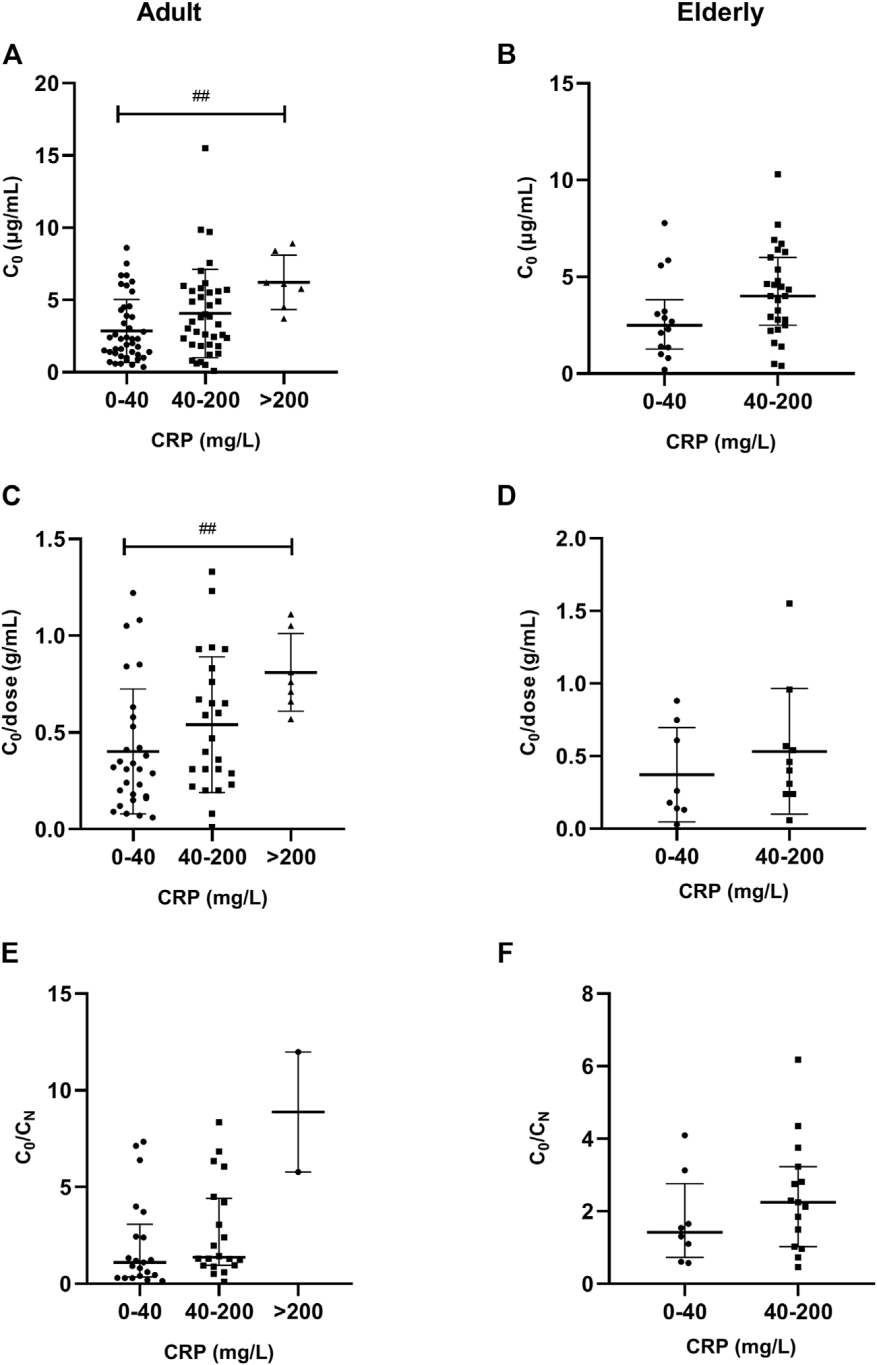
Association of CRP plasma levels with VCZ C_0_, C_0_/dose ratio and VCZ C_0_/C_N_ ratio in adult and elderly patients. **(A,B)** Distribution of VCZ C_0_. **(C,D)** Distribution of VCZ C_0_/dose ratio. **(E,F)** Distribution of VCZ C_0_/C_N_ ratio. ^##^ *p* < 0.01.

A significantly (*p* = 0.003) higher VCZ C_0_ was observed in patients with severe inflammation [6.10 (IQR, 5.14–7.30) mg/L; *n* = 7] than in patients with moderate inflammation [3.50 (1.91–5.61) mg/L; *n* = 39] and no to mild inflammation [2.30 (1.25–4.10) mg/L; *n* = 43], as reflected by the CRP level in adult patients; there was a tendency in elderly patients (*p* = 0.06). These differences were shown in Figure 3.

**FIGURE 4:**
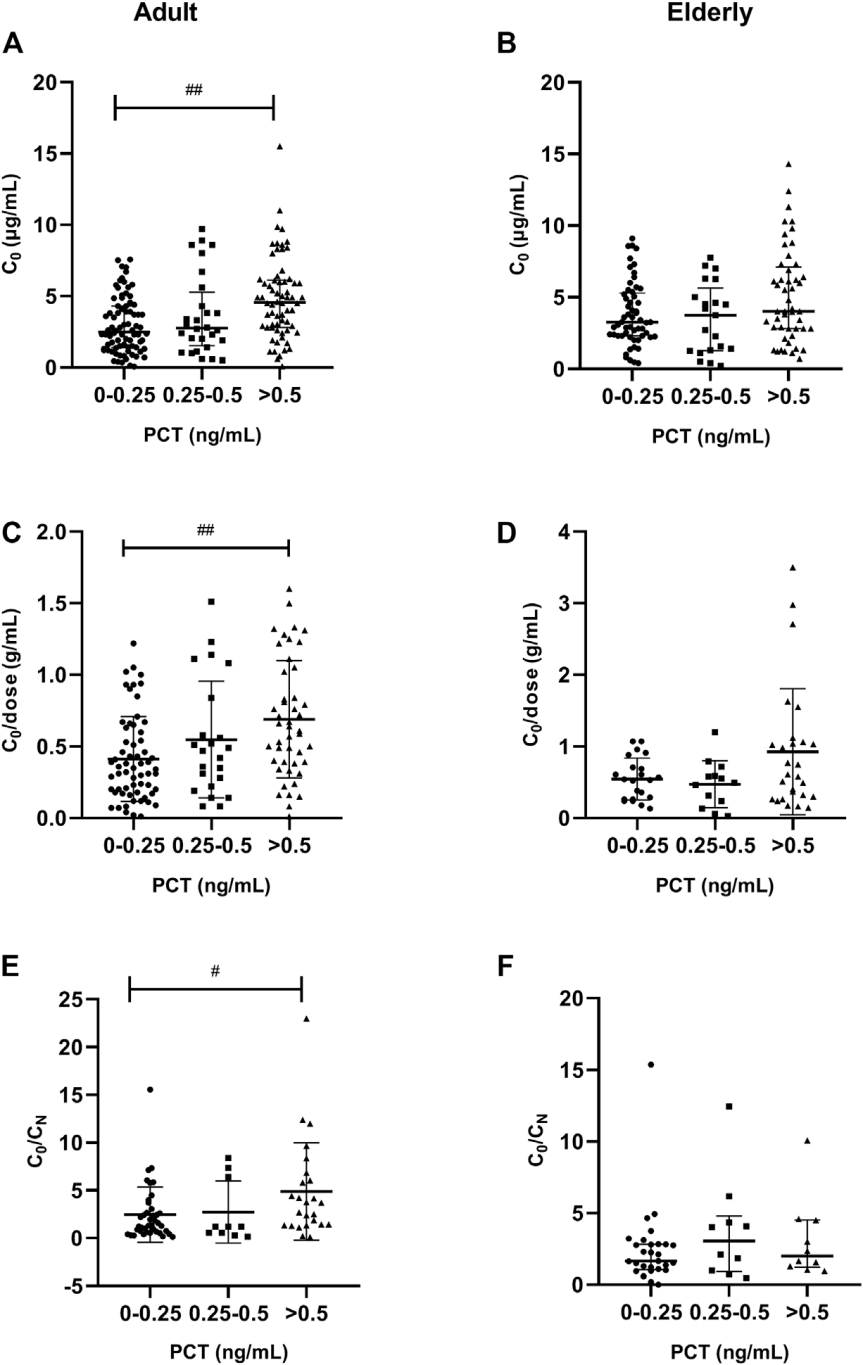
Association of PCT plasma levels with VCZ C_0_, C_0_/dose ratio and VCZ C_0_/C_N_ in adult and elderly patients. **(A,B)** Distribution of VCZ C_0_. **(C,D)** Distribution of VCZ C_0_/dose ratio. **(E,F)** Distribution of VCZ C_0_/C_N_ ratio. ^#^ *p* < 0.05, ^##^ *p* < 0.01.

A significantly (*p* < 0.001) higher VCZ C_0_ was also observed in patients with severe inflammation [4.60 (IQR, 2.90–6.10) mg/L; *n* = 66] than in patients with moderate inflammation [2.70 (1.40–4.30) mg/L; *n* = 28] and no to mild inflammation [2.49 (1.40–4.30) mg/L; *n* = 80], as reflected by the PCT level in adult patients; there was a tendency in elderly patients (*p* = 0.068). These differences were shown in Figure 4.

### Correlations Between Inflammatory Markers and VCZ C_0_/Dose Ratio

In adult patients, CRP and IL-6 levels showed shallow association with VCZ C_0_/dose ratio (CRP: r = 0.326, *p* = 0.010, 61 observations; IL-6: r = 0.370, *p* < 0.001, 86 observations). There was no association between inflammatory biomarkers and VCZ C_0_/dose ratio in elderly patients.

A significantly (*p* = 0.010) higher VCZ C_0_/dose ratio was observed in patients with severe inflammation [0.76 (0.69–0.93) g/ml; *n* = 7] than in patients with moderate inflammation [0.47 (0.29–0.76) g/ml; *n* = 25] and no to mild inflammation [0.31 (IQR, 0.17–0.53) g/ml; *n* = 29], as reflected by the CRP level in adult patients. There was no significant difference observed among different inflammation degrees in elderly patients (Figure 3).

A significantly (*p* = 0.001) higher VCZ C_0_/dose ratio was also observed in patients with severe inflammation [0.63 (IQR, 0.39–0.97) g/ml; *n* = 46] than in patients with moderate inflammation [0.47 (0.25–0.71) g/ml; *n* = 23] and no to mild inflammation [0.35 (0.19–0.58) g/ml; *n* = 62], as reflected by the PCT level in adult patients. There was no significant difference observed among different inflammation degrees in elderly patients (Figure 4).

### Correlations Between Inflammatory Markers and VCZ C_0_/C_N_ Ratio

In adult patients, CRP and IL-8 levels showed moderate association with VCZ C_0_/C_N_ ratio (CRP: r = 0.512, *p* < 0.001, 45 observations; IL-8: r = 0.476, *p* = 0.002, 40 observations). PCT and IL-6 had shallow association with VCZ C_0_/C_N_ ratio (PCT: r = 0.329, *p* = 0.004, 75 observations; IL-6: r = 0.355, *p* = 0.003, 67 observations) (Figure 1). In elderly patients, only IL-6 had shallow association with VCZ C_0_/C_N_ ratio (IL-6: r = 0.386, *p* = 0.001, 67 observations) (Figure 2).

In adult patients, a significantly (*p* = 0.025) higher VCZ C_0_/C_N_ ratio was observed in patients with severe inflammation [4.00 (IQR, 2.80–6.90) mg/L; *n* = 25] than in patients with moderate inflammation [3.22 (1.29–4.70) mg/L; *n* = 10] and no to mild inflammation [3.27 (2.30–5.34) mg/L; *n* = 39], as reflected by the PCT level; however, a tendency of significant association was observed, as reflected by the CRP level (*p* = 0.074). These differences were shown in Figure 4. In elderly patients, there was no significant difference in C_0_/C_N_ ratio under different inflammation degrees, as reflected by CRP or PCT levels.

### Liver Function Results

**FIGURE 5:**
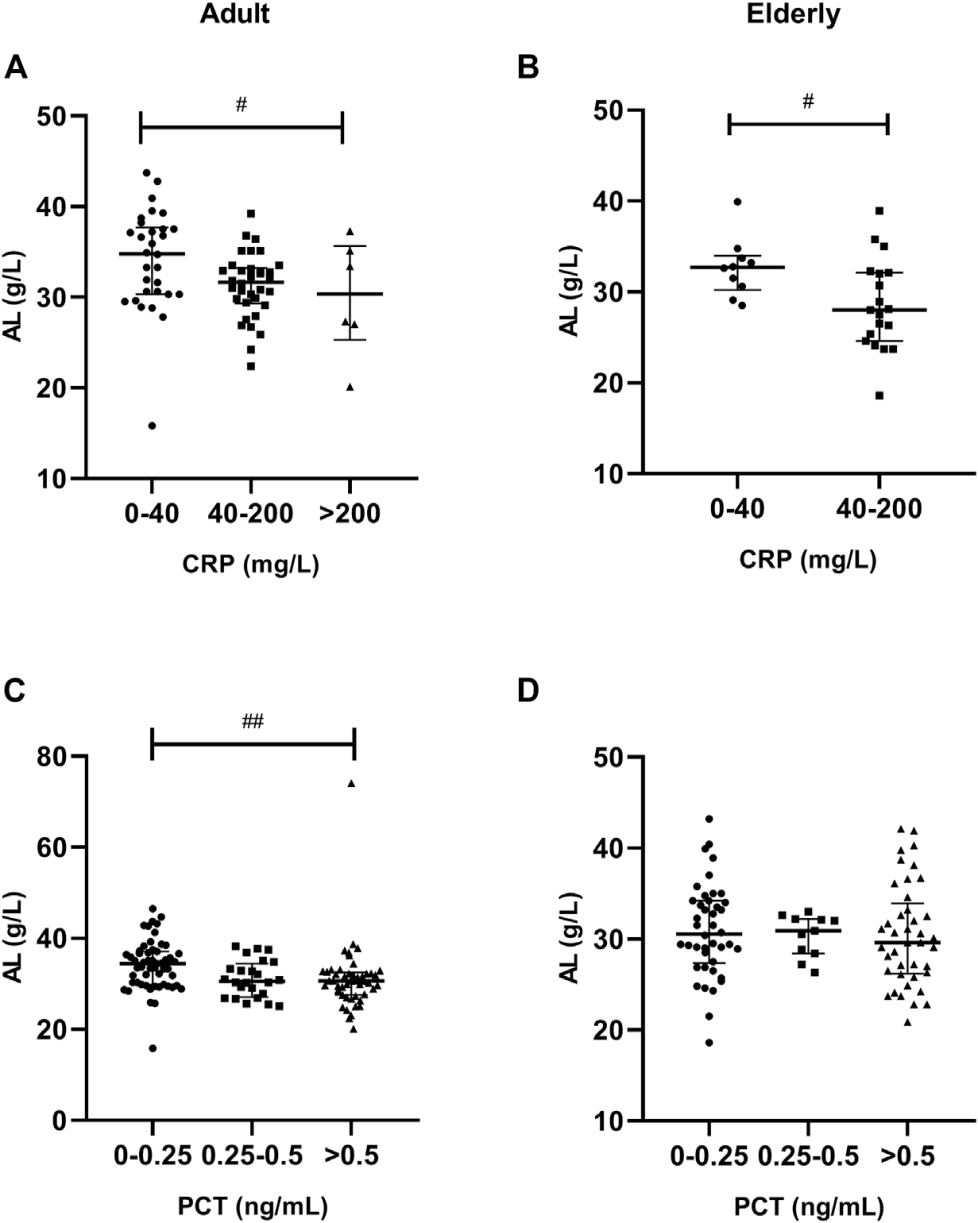
Association of CRP and PCT plasma levels with albumin (AL) in adult and elderly patients. **(A,B)** Distribution of AL according to CRP. **(C,D)** Distribution of AL according to PCT. ^#^ *p* < 0.05, ^##^ *p* < 0.01.

**FIGURE 6:**
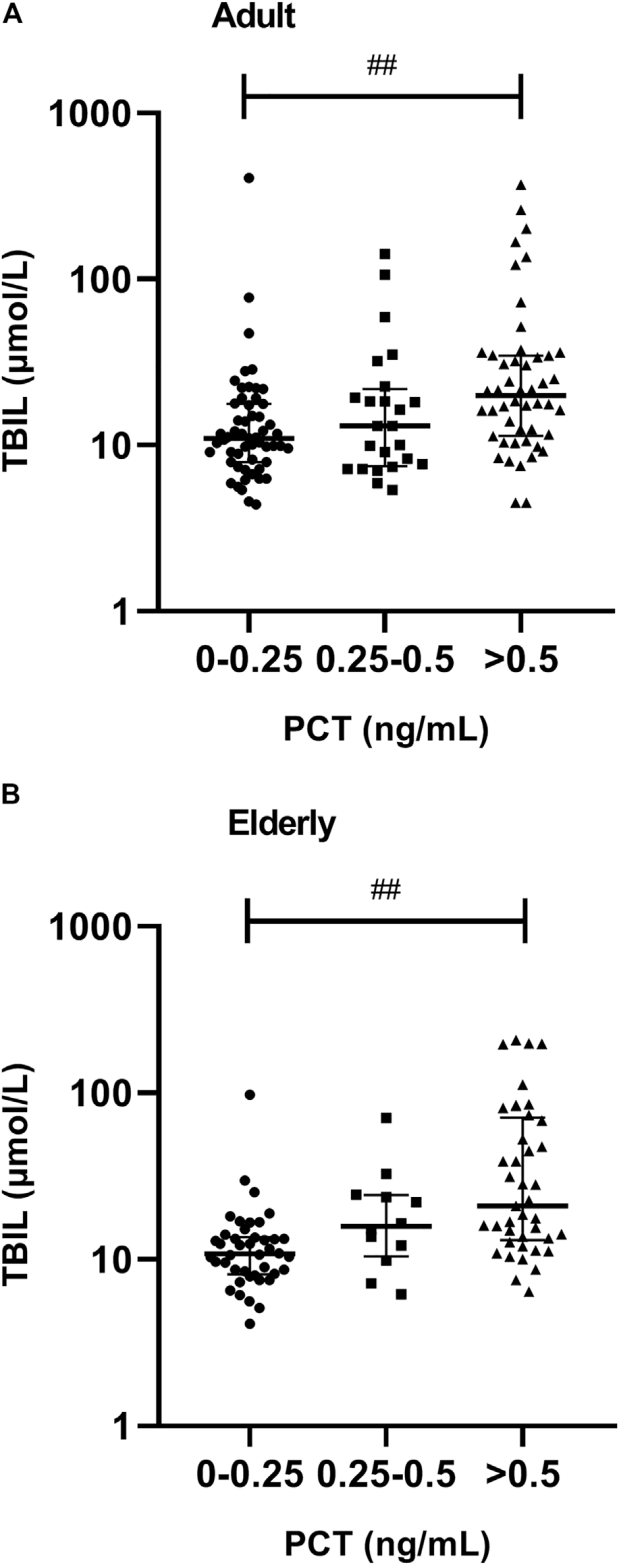
Association between PCT plasma levels and total bilirubin (TBIL) in adult and elderly patients. **(A)** Distribution of TBIL in adult patients. **(B)** Distribution of TBIL in elderly patients. ^##^ *p* < 0.01.

A significantly lower AL was observed in patients with severe inflammation than in patients with moderate inflammation and no to mild inflammation among adult patients, both reflected by CRP and PCT levels (*p* < 0.05), and the association was obvious in elderly patients, as reflected by CRP levels (*p* = 0.001) (Tables 3, 4; Figure 5). A significantly higher TBIL was observed in patients with severe inflammation than in patients with moderate inflammation and no to mild inflammation in both adult and elderly cohorts, as reflected by the PCT level (*p* < 0.01) (Table 4; Figure 6).

**TABLE 3:** Comparisons of liver function under different inflammation degrees as reflected by CRP levels in the two cohorts.

Cohort	Liver function	CRP (mg/ml)			*p* value
		0–40	40–200	200-	
Adult cohort	ALP (U/L)	93.0 (78.3, 128.0)	99.5 (74.3, 153.8)	79.5 (51.0, 87.8)	0.173
	ALT (U/L)	19.9 (15.5, 42.1)	18.7 (8.2, 43.9)	11.0 (8.4, 13.6)	0.157
	AST (U/L)	22.4 (19.8, 43.2)	22.3 (16.7, 51.8)	16.7 (13.4, 21.3)	0.112
	γ-GT (U/L)	79.3 (55.1, 183.9)	69.2 (35.0, 112.5)	41.6 (28.3, 76.5)	0.255
	TBIL (μmol/L)	12.0 (9.3, 19.2)	15.5 (9.0, 22.3)	22.6 (13.1, 30.0)	0.441
	AL (g/L)	34.8 (30.4, 37.5)	31.7 (29.5, 33.1)	30.4 (27.1, 34.7)	0.034
Elderly cohort	ALP (U/L)	80.0 (57.0, 110.0)	97.0 (71.0, 125.0)	—	0.302
	ALT (U/L)	15.9 (12.6, 38.2)	28.0 (14.4, 48.6)	—	0.591
	AST (U/L)	25.8 (23.7, 57.9)	34.2 (20.9, 68.2)	—	0.983
	γ-GT (U/L)	82.0 (31.6, 153.4)	50.0 (42.9, 114.1)	—	0.561
	TBIL (μmol/L)	9.9 (9.1, 13.9)	13.1 (10.6, 16.8)	—	0.149
	AL (g/L)	32.7 (30.8, 33.6)	28.0 (25.0, 32.1)	—	0.017

“-”, not available; data are presented as median (interquartile range). ALP, alkaline phosphatase; ALT, alanine aminotransferase; AST, aspartate aminotransferase; γ-GT, γ-glutamyl transferase; TBIL, total bilirubin; AL, albumin.

**TABLE 4:** Comparisons of liver function under different inflammation degrees as reflected by PCT levels in the two cohorts.

Cohort	Liver function	PCT (ng/ml)			*p* value
		0–0.25	0.25–0.5	0.5-	
Adult cohort	ALP (U/L)	88.2 (72.5, 125.0)	93.0 (74.0, 153.0)	92.0 (68.0, 165.0)	0.616
	ALT (U/L)	22.8 (12.6, 42.4)	13.3 (8.1, 30.3)	13.1 (8.1, 35.8)	0.080
	AST (U/L)	21.9 (17.2, 36.9)	19.6 (14.3, 49.7)	21 (14.3, 71.0)	0.686
	γ-GT (U/L)	78.6 (34.7, 187.5)	75.2 (46.2, 158)	67.1 (31.9, 128.3)	0.829
	TBIL (μmol/L)	11.0 (8.1, 17.6)	13.1 (7.7, 19.3)	21.2 (12.1, 34.6)	0.001
	AL (g/L)	34.4 (29.8, 37.0)	30.6 (27.8, 33.3)	30.7 (27.6, 32.3)	0.001
Elderly cohort	ALP (U/L)	103.0 (74.0, 124.8)	156.0 (65.5, 184.8)	129.0 (82.0, 227.0)	0.140
	ALT (U/L)	22.0 (11.2, 34.8)	15.5 (10.0, 24.4)	24.6 (8.5, 72.9)	0.513
	AST (U/L)	30.6 (19.8, 46.1)	25.1 (22.7, 35.5)	38.6 (25.8, 80.6)	0.084
	γ-GT (U/L)	52.6 (33.8, 124.5)	146.0 (50.7, 188.5)	67.0 (42.5, 168.5)	0.218
	TBIL (μmol/L)	10.8 (8.3, 13.4)	15.8 (11.5, 23.8)	20.9 (13.4, 68.1)	0.000
	AL (g/L)	30.6 (27.8, 34.2)	30.9 (28.6, 32.2)	29.6 (26.3, 33.2)	0.712

Data are presented as median (interquartile range). ALP, alkaline phosphatase; ALT, alanine aminotransferase; AST, aspartate aminotransferase; γ-GT, γ-glutamyl transferase; TBIL, total bilirubin; AL, albumin.

## Discussion

In the present study, we examined common inflammation markers, such as CRP, PCT, and IL-6, which were detected in the clinic, and four other cytokines, both pro- and anti-inflammatory, for their correlation with VCZ C_0_, C_0_/dose ratio and VCZ C_0_/C_N_ ratio. CRP has been mostly investigated to be associated with VCZ C_0_ in hematologic malignancy and solid organ transplant patients (42–62 years) (van Wanrooy et al., 2014; Encalada Ventura et al., 2016; Vreugdenhil et al., 2018), and CRP levels are the main risk factor for VCZ overdose in French hematological patients (Gautier-Veyret et al., 2019).

In a prospective single-centre analysis performed in adult haematology patients receiving VCZ, which included 22 patients and 143 samples, the CRP, IL-6, and IL-8 levels were significantly associated with the VCZ C_0_ (CRP: r = 0.53, *p* < 0.0001; IL-6: r = 0.53, *p* < 0.0001; IL-8: r = 0.42, *p* < 0.0001) (Vreugdenhil et al., 2018). In a retrospective chart review of VCZ, a total of 77 samples from 63 patients were obtained, and the CRP level was significantly associated with the VCZ C_0_/dose ratio (r^2^ = 0.26, *p* < 0.001) (Dote et al., 2016). In our study, 195 patients with 298 samples in the adult cohort and 161 patients with 255 samples in the elderly cohort were included, the correlation coefficient of CRP with VCZ C_0_ was 0.289 and 0.370 in adult and elderly patients, respectively. The low correlation coefficient may be due to 1-2 samples from one patient in our study, while 6-7 samples from one patient in the prospective study. The retrospective nature of the study and the large number of patients may contribute to the increased sample distribution widths.

Niioka et al., 2017 investigated the relationship of the CYP2C19 phenotype with the VCZ C_0_/C_N_ ratio and demographic and clinical characteristics of Japanese patients, and found that patients with intravenous administration, higher CRP on the same day of VCZ plasma concentration measurement, CYP2C19 extensive metabolizer, and old age had higher VCZ C_0_/C_N_ ratio. In our study, only 7.7% (adult) and 5.0% (elderly) patients had oral administration; the median CRP level was 42.90 in adult patients and 64.07 in elderly patients, respectively; the median value of C_0_/C_N_ ratio was 1.42 in adult patients and 2.05 in elderly patients, and the VCZ C_0_/C_N_ ratio in elderly patients was significantly higher than that in adult patients, which were consistent with previous study.

CRP concentrations was reported to significantly influence the VCZ C_0_/C_N_ ratio even when corrected for other factors that could influence VCZ metabolism in a prospective observational study (Veringa et al., 2017). In addition to CRP level being significantly associated with VCZ C_0_/C_N_ ratio in adult patients, our results also showed that IL-8 plasma levels were moderately associated with VCZ C_0_/C_N_ ratio in adult patients, and IL-6 plasma levels were shallowly associated with VCZ C_0_/C_N_ ratio in adult and elderly patients, which has not been reported previously. In an inflammatory state, LPS, TNF-α, IL-1β, and IL-6 can bind to cytokine receptors and TLR4 receptors on the cell membrane, regulating the expression of transporters and drug-related metabolic enzymes by targeting nuclear receptors, liver X receptor (LXR), pregnane X receptor (PXR) and constitutive rostane receptor (CAR), through the NF-κB signaling pathway (Wu and Lin, 2019). Furthermore, IL-6 can directly target liver cells and down regulate CYP2C19 and CYP3A4 gene expression during inflammation (Li et al., 2014; Klein et al., 2015). In particular, IL-6 caused a 30%–50% reduction in CYP2C19 mRNA expression in human hepatocytes (Aitken and Morgan, 2007; Rieger et al., 2015). The mechanism by which IL-8 influences the metabolism of VCZ is not clear. In the early stage of liver injury, IL-8 recruits neutrophils to the injured tissue through its specific receptors CXCR1 and CXCR2 and adheres to liver cells through intracellular adhesion factor 1, releasing myeloperoxidase and reactive oxygen species (ROS), resulting in hepatocyte damage (Heymann and Tacke, 2016; Khanam et al., 2017; Li et al., 2017). This may explain the positive correlation between the IL-8 level and VCZ C_0_/C_N_ ratio.

CRP, PCT, and IL-6 were routine inflammatory biomarkers detected in clinic. In the current study, we found that CRP and IL-8 levels were associated with VCZ C_0_/C_N_ ratio in adult patients, while only IL-6 level was shallowly associated with VCZ C_0_/C_N_ ratio in elderly patients, which indicated that IL-6 would be a better marker when evaluating the metabolism of VCZ in the elderly.

VCZ is bound to AL. Decreased AL will increase unbound fraction of VCZ. A positive relationship between VCZ plasma protein binding (PPB) and plasma AL concentrations was observed, indicating higher unbound VCZ concentrations with decreasing AL concentrations (Vanstraelen et al., 2014). Decreased AL levels were also associated with a significantly increased VCZ C_0_/dose ratio (Dote et al., 2016). In patients with invasive aspergillosis, serum AL and γ-GT levels were significantly correlated with the VCZ clearance rate (Chantharit et al., 2020). Association of decreased AL and increased VCZ C_0_ is related to reflection of impaired liver function (changes in intrinsic clearance). In our previous study, we determined that decreased AL was significantly associated with increased VCZ C_0_ in adult patients (Cheng et al., 2019). In the present study, we found that, in both adult and elderly patients, a significantly lower AL was observed in patients with severe inflammation than in patients with moderate inflammation and no to mild inflammation, as reflected by CRP levels, which indicated that inflammation induced AL decreasing also contributed to VCZ metabolism.

Apart from the plasma AL concentration, the TBIL plasma concentration also significantly influences VCZ PPB (Vanstraelen et al., 2014). The presence of a bilirubin value of >1.5 mg/dL was associated with supratherapeutic concentrations in critically ill patients (Ruiz et al., 2019). In invasive fungal infection patients with liver dysfunction, VCZ clearance was significantly associated with TBIL and platelet count (Tang et al., 2020). Our previous study also determined that increased TBIL was significantly associated with increased VCZ C_0_ in adult patients (Cheng et al., 2019). The results of the current study showed that, in both adult and elderly patients, significantly increased TBIL was observed in patients along with the severity of inflammation, as reflected by PCT levels. The decreased AL level and increased TBIL level along with the degree of inflammation provided another explanation for the increased VCZ C_0_ under the inflammatory state.

This study has several limitations. We do not determine VCZ C_N_ in feces and urine specimens. A ratio of <2% VCZ is excreted through renal as the parent drug (Roffey et al., 2003; Geist et al., 2013; Hohmann et al., 2017). Geist et al. found that VCZ N-oxide and its conjugates excreted in urine within 12 h post dose during steady-state only accounted for 1% of the dose (Geist et al., 2013). In the current study, we also determine the C_0_ and C_N_ of VCZ at 12 h post drug administration during steady-state, and we propose that the C_N_ in feces and urine specimens may be rare. We previously demonstrated that VCZ C_0_ and C_0_/C_N_ ratio were influenced by CYP2C19 polymorphisms in adult patients, but the influence was not obvious in elderly patients (Shang et al., 2020). However, the adult cohort was not stratified by CYP2C19 polymorphism status in the current analysis for its retrospective nature. Some other liver biomarkers such as ALP, ALT, AST, and γ-GT have been investigated to be associated with VCZ C_0_, changes of these biomarkers reflect the function of liver, thus affecting VCZ metabolism. In the current study, we focus on the effect of inflammation on liver function and VCZ metabolism, and find that ALP, ALT, AST, and γ-GT are not significantly influenced by inflammation.

In conclusion, this study clearly demonstrates that inflammation, reflected by IL-6 and IL-8, may affect the metabolism of VCZ to VCZ-N-oxide in both adult and elderly patients. Furthermore, the decreased plasma AL levels and increased plasma TBIL levels under inflammatory conditions may also alter VCZ metabolism. The inflammation state and impaired liver function should be considered when optimizing VCZ doses to decrease the risk of adverse effects in elderly.

## Data Availability

The original contributions presented in the study are included in the article/Supplementary Material, further inquiries can be directed to the corresponding authors.
